# Supplementary material for: A Critical View of the Application of the APEX Software (Aqueous Photochemistry of Environmentally-Occurring Xenobiotics) to Predict Photoreaction Kinetics in Surface Freshwaters
Source: Molecules. 2019 Dec 18;25(1):9. doi: 10.3390/molecules25010009 (PMC7017383; doi:10.3390/molecules25010009)
Supplement: Supplementary file 1 [file molecules-25-00009-s001.zip › 3.2.4_gcc-4.4.0.zip!/3.2.4_gcc-4.4.0/3.2.4_gcc-4.4.0/mingw32/doc/dos2unix/dos2unix.html]

Content-type: text/html
Manpage of dos2unix

# dos2unix

Section: User Commands (1)  
Updated: dos2unix v3.0  
Index
Return to Main Contents

---

 

## NAME

dos2unix - DOS/MAC to UNIX text file format converter

 

## SYNOPSYS

dos2unix [options] [-c convmode] [-o file ...] [-n infile outfile ...]

Options:

[-hkqV] [--help] [--keepdate] [--quiet] [--version]

 

## DESCRIPTION

This manual page documents dos2unix, the program that converts plain text
files in DOS/MAC format to UNIX format.

 

## OPTIONS

The following options are available:

**-h --help**: Print online help. **-k --keepdate**: Keep the date stamp of output file same as input file. **-q --quiet**: Quiet mode. Suppress all warning and messages. **-V --version**: Prints version information. **-c --convmode convmode**: Sets conversion mode. Simulates dos2unix under SunOS. **-o --oldfile file ...**: Old file mode. Convert the file and write output to it. The program default to run in this mode. Wildcard names may be used. **-n --newfile infile outfile ...**: New file mode. Convert the infile and write output to outfile. File names must be given in pairs and wildcard names should NOT be used or you WILL lost your files.

 

## EXAMPLES

Get input from stdin and write output to stdout.

: **dos2unix**

Convert and replace a.txt. Convert and replace b.txt.

: **dos2unix a.txt b.txt** : **dos2unix -o a.txt b.txt**

Convert and replace a.txt in ASCII conversion mode.
Convert and replace b.txt in ISO conversion mode.
Convert c.txt from Mac to Unix ascii format.

: **dos2unix a.txt -c iso b.txt** : **dos2unix -c ascii a.txt -c iso b.txt** : **dos2unix -c mac a.txt b.txt**

Convert and replace a.txt while keeping original date stamp.

: **dos2unix -k a.txt** : **dos2unix -k -o a.txt**

Convert a.txt and write to e.txt.

: **dos2unix -n a.txt e.txt**

Convert a.txt and write to e.txt, keep date stamp of e.txt same as a.txt.

: **dos2unix -k -n a.txt e.txt**

Convert and replace a.txt. Convert b.txt and write to e.txt.

: **dos2unix a.txt -n b.txt e.txt** : **dos2unix -o a.txt -n b.txt e.txt**

Convert c.txt and write to e.txt. Convert and replace a.txt.
Convert and replace b.txt. Convert d.txt and write to f.txt.

: **dos2unix -n c.txt e.txt -o a.txt b.txt -n d.txt f.txt**

 

## DIAGNOSTICS

 

## BUGS

The program does not work properly under MSDOS in stdio processing mode.
If you know why is that so, please tell me.

 

## AUTHORS

Benjamin Lin -
**<blin@socs.uts.edu.au>**

Bernd Johannes Wuebben (mac2unix mode)
**<wuebben@kde.org>**

 

## MISCELLANY

Tested environment:

: Linux 1.2.0 with GNU C 2.5.8 : SunOS 4.1.3 with GNU C 2.6.3 : MS-DOS 6.20 with Borland C++ 4.02

Suggestions and bug reports are welcome.

 

## SEE ALSO

unix2dos(1) mac2unix(1)

---

 

## Index

NAME: SYNOPSYS: DESCRIPTION: OPTIONS: EXAMPLES: DIAGNOSTICS: BUGS: AUTHORS: MISCELLANY: SEE ALSO

---

This document was created by
man2html,
using the manual pages.  
Time: 11:51:18 GMT, December 04, 2002
